# Bronchoalveolar Activation of Coagulation and Inhibition of Fibrinolysis during Ventilator-Associated Lung Injury

**DOI:** 10.1155/2012/961784

**Published:** 2012-04-17

**Authors:** M. J. Schultz, R. M. Determann, A. A. N. M. Royakkers, E. K. Wolthuis, J. C. Korevaar, M. M. Levi

**Affiliations:** ^1^Department of Intensive Care Medicine, Academic Medical Center, Meibergdreef 9, Amsterdam, 1105 AZ, The Netherlands; ^2^Department of Internal Medicine, Academic Medical Center, Meibergdreef 9, Amsterdam, 1105 AZ, The Netherlands; ^3^Department of Anesthesiology, Tergooi Ziekenhuizen, Rijksstraatweg 1, Blaricum, 1261 AN, The Netherlands; ^4^Department of Anesthesiology, Academic Medical Center, Meibergdreef 9, Amsterdam, 1105 AZ, The Netherlands; ^5^Department of Biostatistics and Epidemiology, Academic Medical Center, Meibergdreef 9, Amsterdam, 1105 AZ, The Netherlands

## Abstract

*Background and Objective*. Bronchoalveolar coagulopathy is a characteristic feature of pulmonary inflammation. We compared bronchoalveolar and systemic levels of coagulation in patients who did and patients who did not develop ventilator-associated lung injury (VALI). *Methods*. Secondary analysis of a randomized controlled trial evaluating the effect of lower tidal volumes versus conventional tidal volumes in patients without acute lung injury or acute respiratory distress syndrome at the onset of mechanical ventilation. *Results*. Ten patients with VALI and 10 random control patients without lung injury during the course of mechanical ventilation, but all ventilated with conventional tidal volumes, were compared. Patients who developed VALI showed both bronchoalveolar activation of coagulation (increase in thrombin–antithrombin complex levels *P* < 0.001 versus baseline) and inhibition of fibrinolysis (decline in plasminogen activator activity *P* < 0.001 versus baseline). The later seemed to be dependent on higher levels of plasminogen activator inhibitor type 1 (*P* = 0.001 versus baseline). Patients who developed VALI also showed elevated systemic thrombin-antithrombin complex levels and decreased systemic plasminogen activator activity levels. *Conclusions*. VALI is characterized by bronchoalveolar coagulopathy. Systemic and bronchoalveolar coagulopathy at the onset of mechanical ventilation may be a risk factor for developing VALI in patients ventilated with conventional tidal volumes.

## 1. Introduction

There is indisputable evidence that mechanical ventilation has the potential to aggravate lung injury. Indeed, two randomized controlled clinical trials confirm the existence of so-called ventilator-associated lung injury (VALI) in patients with acute lung injury or acute respiratory distress syndrome (ALI/ARDS) by showing reduced morbidity and mortality with lung-protective mechanical ventilation using lower tidal volumes [1, 2]. Current guidelines, therefore, now support the use of lower tidal volumes in patients with ALI/ARDS [3].

There is increasingly more evidence supporting the use of lower tidal volume in critically ill patients without ALI/ARDS. Nonprotective forms of mechanical ventilation potentiate the local inflammatory response in patients with ALI/ARDS via local upregulation of proinflammatory mediators [4, 5]. Several small studies, mainly focusing on the effects of short-term mechanical ventilation in the operating theater, have extended these data to patients not suffering from ALI/ARDS. Nonprotective mechanical ventilation settings result in procoagulant activity in the pulmonary compartment and systemic upregulation of proinflammatory cytokines, although the results of studies were not always consistent [6]. Results from recent cohort studies suggest that mechanical ventilation with conventional tidal volumes may cause or contribute to development of VALI in critically ill patients who do not have ALI/ARDS at the onset of mechanical ventilation [7, 8]. Finally, we recently stopped a randomized controlled trial comparing conventional tidal volumes with lower tidal volumes in patients without ALI/ARDS at onset of mechanical ventilation prematurely because development of VALI during the course of mechanical ventilation was higher in the group treated with conventional tidal volumes as compared to the lower tidal volume group [9].

Alveolar fibrin deposition is an important feature of pulmonary inflammation. The mechanisms that contribute to disturbed bronchoalveolar fibrin turnover are localized tissue factor- (TF-) mediated thrombin generation and depression of fibrinolysis, caused by the increase of inhibitors of the plasminogen activator, in particular plasminogen activator inhibitor type 1 (PAI-1) [10]. These effects on pulmonary coagulation and fibrinolysis are regulated by various proinflammatory cytokines and are similar to those found in the intravascular spaces during severe systemic inflammation. We showed before that the use of conventional tidal volumes during a short course of mechanical ventilation because of a surgical procedure also causes pulmonary coagulopathy [11]. Ventilator-induced coagulopathy in this study was largely prevented with the use of lower tidal volumes.

In the present analysis, we performed a secondary analysis of the recently performed clinical trial on conventional versus lower tidal volumes in patients without ALI/ARDS. In the present analysis we compared patients who did and patients who did not develop VALI in the conventional tidal volume arm of that study, with respect to bronchoalveolar and systemic coagulopathy. Our aim was to determine whether development of VALI with the use of conventional tidal volumes is characterized by pulmonary coagulopathy alike the use of conventional tidal volumes with short-term mechanical ventilation.

## 2. Patients and Methods

### 2.1. Patients

In the original study 150 patients were included at the intensive care units of one regional teaching hospital and one academic in The Netherlands. Patients were eligible if they did not meet the North-American European Consensus Conference criteria for ALI/ARDS [12] and needed mechanical ventilation for an anticipated duration of at least 3 days as described previously. Randomization, data and sample collection had to be started within 36 hours of initiation of mechanical ventilation. Exclusion criteria were: age under 18 years, participation in other clinical trials, pregnancy, increased uncontrollable intracranial pressure, history of severe chronic obstructive pulmonary disease or severe restrictive pulmonary disease, history of pneumectomy or lobectomy, use of immunosuppressive agents (>100 mg hydrocortisone per day), pulmonary thromboembolism, and previous randomization in this study. The protocol was approved by the medical ethics committee of both hospitals, and written informed consent was obtained from the patient or closest relative before the start of the study.

### 2.2. Mechanical Ventilation

The study protocol was as described previously. In short, the volume control mode was used for mechanical ventilation. The target tidal volume was calculated as 10 or 6 mL times the predicted body weight as described previously. The inspiratory flow rate was maintained at 40 liters per minute. As soon as patients were able to breathe spontaneously with assistance, the pressure support mode was used. In this case, the pressure support was adjusted in order to reach the target tidal volume. As soon as patients were ready to be weaned from the ventilator the amount of support was decreased at discretion of the attending physician. If a patient met the North-American European Consensus Conference criteria [12] for ALI/ARDS, the attending intensive care physician was advised to ventilate the patient with a lung-protective mechanical ventilation strategy allowing tidal volumes of 6 mL per kilogram predicted body weight in the pressure control mode (according to the local institutional protocol for mechanical ventilation in patients with ALI/ARDS) for the remaining ventilation period.

### 2.3. Data Collection

Demographic data, ventilation parameters, and clinical and radiological data were recorded each second day, for calculation of acute physiology and chronic health evaluation- (APACHE-) II score, sequential organ failure assessment (SOFA) score, and lung injury score (LIS). In addition, blood gas parameters were recorded until the patient was weaned from the ventilator.

On the day of enrollment and each second day until the patient was weaned from the ventilator, a bronchoalveolar minilavage was performed for measurement of levels of coagulation and fibrinolysis. Minilavage was performed as described previously [13]. In short, a 20 mL syringe was filled with 0.9% saline and attached to a 50 cm 14 Fr tracheal suction catheter. The catheter was inserted in the orotracheal tube and advanced until significant resistance was encountered. Saline was instilled over 10 seconds and immediately aspirated. The recovery was 4–8 mL in general. For coagulation assays, EDTA was added to the lavage fluids to a final concentration of 1.7 mM. The recovered fluid was centrifuged at 1,500 ×g for 10 minutes at 4°C. The supernatant was collected and stored at −80°C until measurements were performed. Citrated (0.109 M) blood samples were drawn prior to each lavage. Samples were centrifuged at 1,500 ×g for 10 minutes at 4°C; supernatants were stored at –80°C.

### 2.4. Measurements

Thrombin-antithrombin complex (TATc), TF, and PAI-1 were measured using specific commercially available ELISA's according to the instructions of the manufacturer (TATc: Behringwerke AG, Marburg, Germany; TF: American Diagnostics, Greenwich, CT, USA; PAI-1: TintElize PAI-1, Biopool, Umea, Sweden). The levels of factor VIIa were determined by using previously described enzyme capture assay for determining factor (F) VIIa activity [14]. Briefly, solid-phase bound monoclonal antibodies rose against recombinant FVIIa enabled capturing of FVIIa. In the next step, bound FVIIa was allowed to convert a fluorogenic substrate during incubation, which is linearly correlated with FVIIa concentrations. Plasminogen activator activity (PAA) was measured by an amidolytical assay [15]. Briefly, 25 *μ*L of plasma was mixed to a final volume of 250 *μ*L with 0.1 M TrisHCl, pH 7.5, 0.1% (v/v) Tween-80, 0.3 mM S-2251 (Chromogenix, Mölndal, Sweden), 0.13 M plasminogen and 0.12 mg/mL CNBr fragments of fibrinogen (Chromogenix, Mölndal, Sweden). The results are expressed as IU/mL. 

### 2.5. Statistical Analysis

The original study was stopped after the second interim analysis as the incidence of ALI/ARDS after randomization was significantly higher in the conventional tidal volume group. By that time 74 patients were randomized to the conventional tidal volume arm and 76 to the lower tidal volume arm. The aim of the present study was to investigate the procoagulant and fibrinolytic activity in patients who developed VALI. In the analysis we included all patients with VALI from the conventional tidal volume arm (*n* = 10). From the remaining 64 patients without VALI from the conventional tidal volume arm, we included 10 patients as controls. These patients were randomly selected by SPSS.

The required sample size of 10 was calculated from data from our previous investigations on pulmonary hemostasis in patients undergoing elective surgery [11]. To detect differences in bronchoalveolar TATc concentrations in patients with and without VALI at a two-sided significance levels of 5% with a power of 80%, the number of patients to be included in each group was 9.

Data are presented as mean with standard deviation for parametric data or as medians with interquartile range [IQR] for non-parametric data. Baseline comparisons between groups were made by the Student's *t*-test, Mann-Whitney *U* test, Chi-square test, or Fisher exact test where appropriate. Levels of coagulation/fibrinolysis at baseline were analyzed with the Mann-Whitney *U* test. To study changes over time, a linear mixed model was constructed by adding time and randomization group as a factors in the model. The interaction between time and randomization group in this model was used to study differences over time between groups. A two-tailed *P* value <0.05 was considered as statistically significant. Data were analyzed using SPSS, version 14.02 (SPSS Inc, Chicago, IL, USA).

## 3. Results

### 3.1. Patients

Ten patients developed VALI after initiation of mechanical ventilation with tidal volumes of 10 mL/kg predicted body weight. In these patients VALI was diagnosed after 1.6 ± 0.5 days, after which the ventilator was set to achieve lung-protective mechanical ventilation with tidal volumes of 6 mL/kg predicted body weight. Ten patients who did not develop ALI/ARDS served as controls. The baseline characteristics and admission diagnoses are shown in Table 1. Patients who developed VALI were sicker, according to higher SOFA scores, tended to have a higher LIS, and suffered more frequently from sepsis.

### 3.2. Ventilation Parameters (Figure 1)

**Figure 1:**
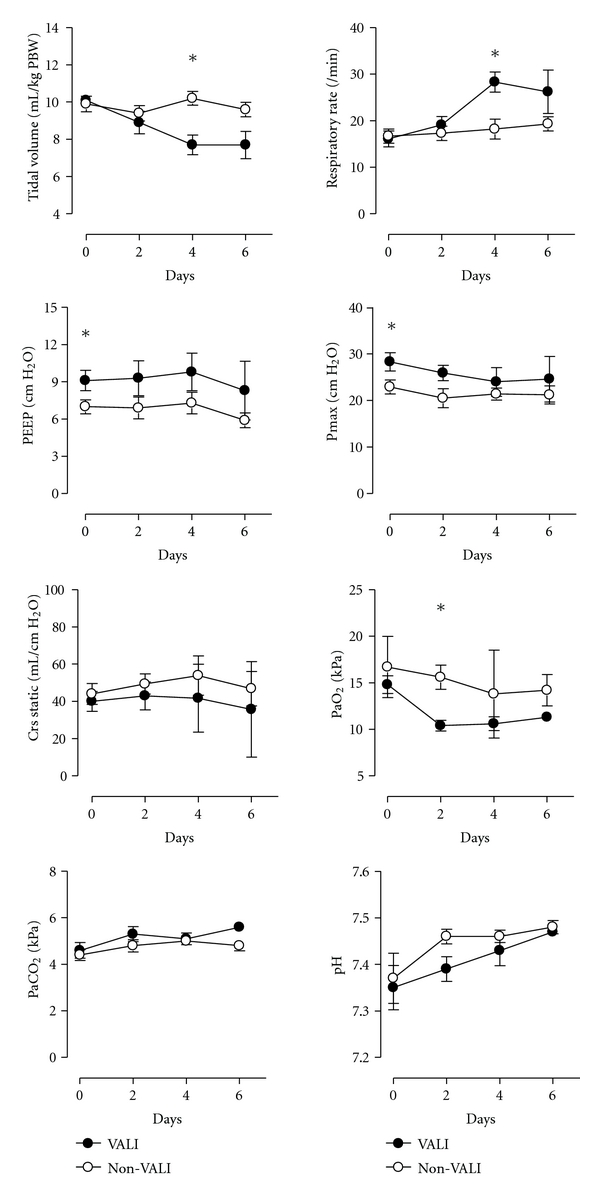
Ventilatory parameters. Tidal volumes per kilogram of predicted body weight (PBW), respiratory rate, positive end expiratory pressures (PEEP), maximum airway pressures (*P*_max⁡_), compliance and blood gas analyses data in patients who developed VALI (closed symbols, *n* = 10), and patients who did not develop VALI (open symbols, *n* = 10). Data are mean ± SD. *P < 0.05.

The period of mechanical ventilation before inclusion was comparable between patients who did and did not develop VALI (22 (14–29) hours versus 14 (10–21) hours, resp.; *P* = 0.13). The applied tidal volumes (10.1 ± 0.7 mL/kg versus 9.9 ± 1.3 mL/kg of predicted body weight; *P* = 0.65; Figure 1), respiratory rate and minute ventilation were comparable at baseline. The tidal volume and respiratory rate were adjusted during the study period in 7 of 10 patients who developed VALI. The patients who developed VALI already had already higher requirements for positive end expiratory pressure (PEEP) at baseline and had concomitant higher maximum airway pressures (Figure 1). The partial oxygen and carbon dioxide pressures and pH were comparable at baseline. The partial oxygen pressure dropped significantly after 2 days (*P* = 0.002) in patients with VALI.

### 3.3. Pulmonary Coagulation and Fibrinolysis

**Figure 2:**
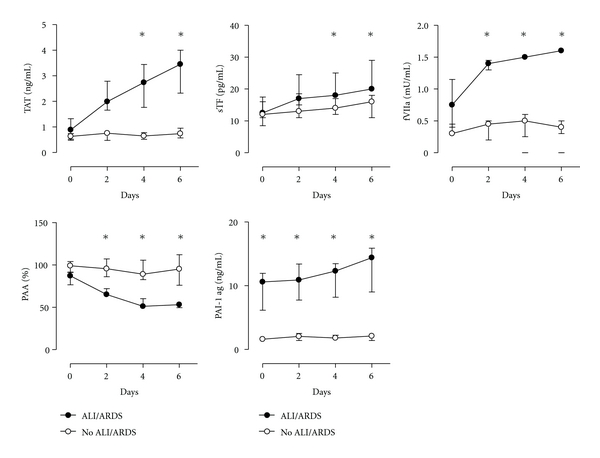
Bronchoalveolar levels of coagulation and fibrinolysis. Thrombin-antithrombin (TATc), soluble tissue factor (TF), factor VIIa (FVIIa), plasminogen activator activity (PAA) and plasminogen activator inhibitor type 1 (PAI-1) in patients who developed VALI (closed symbols, *n* = 10), and patients who did not develop VALI (open symbols, *n* = 10). Data are medians ± IQR. *P < 0.05.

In patients who developed VALI activation of pulmonary coagulation was observed, as reflected in a marked increase in TATc during the course of mechanical ventilation (all *P* < 0.001 versus day 0; Figure 2). TATc levels increased from 0.89 (0.54–1.26) ng/mL at baseline to a maximum of 3.45 (1.76–4.00) ng/mL after 6 days of mechanical ventilation. In contrast, in patients who did not develop VALI during the study pulmonary TATc levels were stable over time (*P* < 0.001 versus VALI patients).

 Thrombin generation appeared to be mediated via the TF/FVIIa pathway, since marked increases in the local levels of soluble TF and FVIIa were measured. Soluble TF levels increased from baseline 12.5 (11–17.3) pg/mL to a maximum of 20.0 (20.0–25.0) pg/mL after 6 days (*P* < 0.001, Figure 2), whereas FVIIa levels showed a 2-fold increase from 0.75 (0.40–1.13) U/mL before the occurrence of VALI to 1.60 (1.50–1.70) mU/mL after 6 days (*P* < 0.001; Figure 2). Local levels of soluble TF and FVIIa in patients who did not develop VALI remained unchanged over time, respectively (*P* = 0.01 and *P* = 0.001 versus VALI patients).

Development of VALI was accompanied by a decreased fibrinolytic activity in lavage fluids. Local plasminogen activator activity (PAA) gradually dropped from 87 (78–91) % to 65 (64–72) % at the day of diagnosis of VALI (*P* < 0.001, Figure 2). PAA levels reached a nadir at 4 days after the diagnosis of VALI. The decrease in PAA in VALI patients appeared to be caused by higher levels of the fibrinolytic inhibitor PAI-1. PAI-1 levels were already higher at baseline 10.6 (6.4–11.9) ng/mL and gradually increased to a maximum of 12.3 (9.0–17.4) ng/mL in patients with VALI (*P* = 0.001). The changes in PAA and PAI-1 were not found in patients who did not develop VALI (*P* = 0.007 and *P* = 0.003 versus VALI patients, resp.).

### 3.4. Systemic Hemostasis (Figure 3)

**Figure 3:**
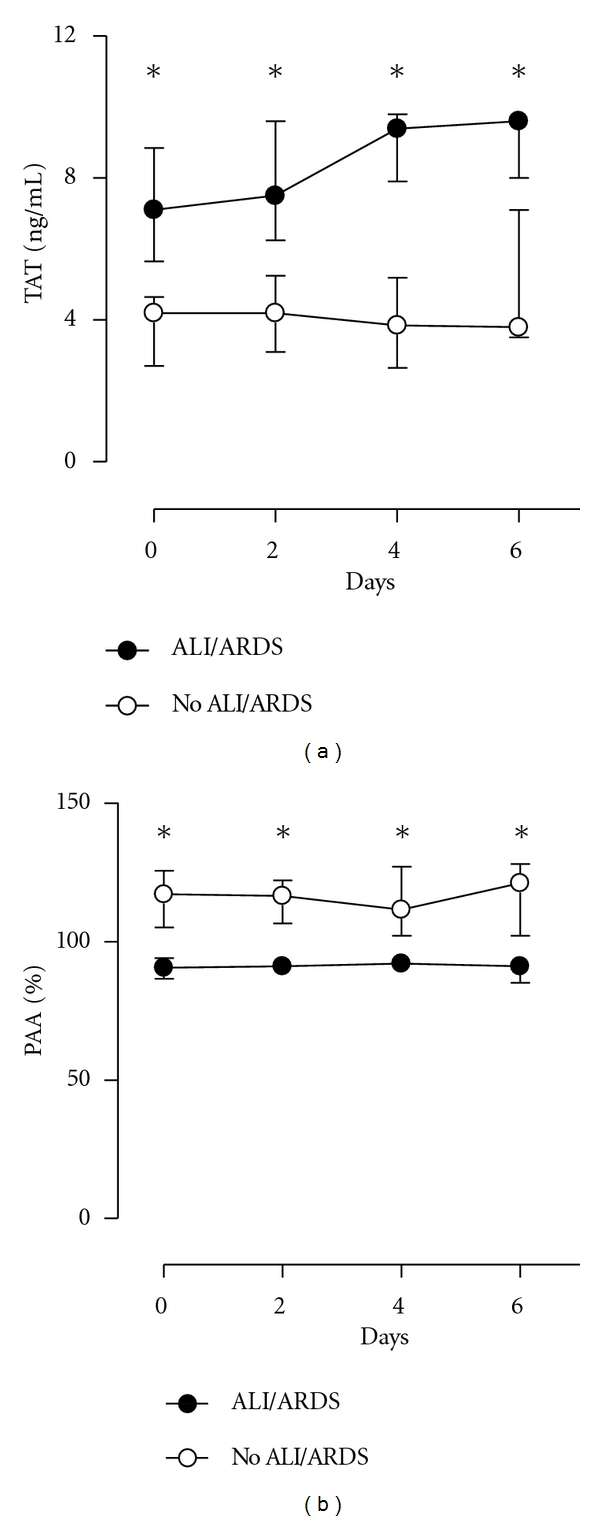
Systemic levels of coagulation and fibrinolysis. Thrombin-antithrombin (TATc) and plasminogen activator activity (PAA) in patients who developed VALI (closed symbols, *n* = 10), and patients who did not develop VALI (open symbols, *n* = 10). Data are medians ± IQR. *, *P* < 0.05.

Plasma TATc levels were higher in patients developing VALI during the course of mechanical ventilation (*P* < 0.001 versus non-VALI patients, Figure 3). In addition, in patients who developed VALI, plasma PAA levels were lower (*P* < 0.001 versus patients no VALI patients). The increase in local TATc levels in patients developing VALI was not accompanied by a significant increase in systemic TATc levels (*P* = 0.35) Figure 3. Plasma PAA-levels remained unchanged during the course of mechanical ventilation (*P* = 0.99).

## 4. Discussion

Although mechanical ventilation with lower tidal volumes is generally considered to be protective in patients with ALI/ARDS, there is ongoing debate on the ideal tidal volumes in patients without preexistent lung injury [16]. We recently showed that the use of conventional tidal volumes, as compared to the use of lower tidal volumes, results in a higher incidence of VALI in patients without ALI/ARDS at the onset of mechanical ventilation [9]. The present study extends previous observation that mechanical ventilation has significant effects on bronchoalveolar hemostasis [11]. Indeed, with development of VALI bronchoalveolar coagulopathy ensues, characterized by activation of coagulation on the one hand and inhibition of fibrinolysis on the other.

Our study has shortcomings. First, we were able to include only a small number of patients. Indeed, only 10 patients in the conventional tidal volume arm of the original study developed VALI [9]. Due to an increased incidence of VALI in the conventional tidal volume arm, the study was discontinued after the second interim analysis. At that moment 150 patients were included and randomized. From an ethical point of view it is impossible to repeat or continue the study, thus we will not be able to make our study population larger. However, the procoagulant changes are sufficiently large enabling us to draw adequate conclusions. Second, it seems that patient selection is not balanced. Indeed, more patients in the VALI group were suffering from sepsis, which is associated with systemic upregulation of coagulation and fibrinolysis parameters [17] and is a well-known risk factor for ALI/ARDS [18]. In the original study, however, patient distribution was very well balanced. Although control patients were randomly selected for this secondary analysis, we are left with this imbalance.

The presently described changes in pulmonary hemostasis are very similar as previously described in patients with pneumonia or ARDS [13, 19, 20], human volunteers with endotoxin-induced pulmonary inflammation [21, 22], and patients ventilated with a nonprotective mechanical ventilation mode during surgery [11]. Consistently, increased procoagulant activity is reported, mostly likely related to the TF-expression on epithelial cells and mononuclear cells in the bronchoalveolar compartment. Also, PAI-1 upregulation has been found consistently in patients with pneumonia or ARDS [13, 19, 20, 23], human volunteers with endotoxin-induced pulmonary inflammation [21] and patients ventilated during surgery with conventional tidal volumes [11]. Although in the settings of endotoxin-induced pulmonary inflammation and short-term mechanical ventilation during surgery, no suppression of fibrinolytic activity was observed, the present study shows that prolonged mechanical ventilation could lead to more interference with fibrinolytic system.

One important observation in the present study is that pulmonary coagulopathy was already present at baseline. Indeed, at baseline bronchoalveolar PAI-1 levels were already increased in patients who developed VALI. This may be related to the fact that 5 patients were septic and therefore had elevated PAI-1 levels in the blood and lungs. Moreover, although not significant, the patients from the VALI group had been ventilated 6 hours longer before randomization. Bronchoalveolar lavage fluid and plasma samples were therefore obtained after a longer period of mechanical ventilation in these patients which may have resulted in higher levels of PAI-1. These patients did not have clinically apparent ALI/ARDS but it is likely that in these patients beginning subclinical lung injury was present at the time of randomization. A “multiple-hit” model of lung injury can be theorized whereby predisposing conditions, like injurious mechanical ventilation, may result in pulmonary inflammation (the “primary hit”). Then, several “second hits” (not per se happening in a consecutive manner, i.e., following the first hit, but maybe or sometimes even likely at the same time), like transfusion of blood products [24], aspiration [2, 25], shock or sepsis [2, 25], and ventilator-associated pneumonia [26], may all cause additional lung injury, finally resulting in full-blown ARDS with high morbidity and mortality.

Earlier studies have shown PAI-1 to be a biomarker of ALI/ARDS. Systemic levels of PAI-1 have been shown to be prognostic in ALI/ARDS patients [27]. The highest levels were observed in patients with the worst outcomes. Moreover, bronchoalveolar PAI-1 levels have also been shown to be diagnostic of ALI/ARDS in patients with aspiration pneumonia [28]. These observations imply that elevated pulmonary PAI-1 levels are indeed associated with lung injury. It is therefore likely that our patients with VALI already had had a first hit that caused local PAI-1 levels to increase.

All of the described effects shift the hemostatic balance towards a procoagulant side, promoting fibrin depositions in the airways. The question remains whether this reflects an adaptive mechanism with host protective functions, or whether it is a harmful process, predisposing the lungs to secondary complications or perhaps long-term effects on pulmonary function. Fibrin deposition may be an important mechanism to repair endothelial or epithelial damage, however an exaggerated coagulation activation has been related to a number of detrimental sequelae.

 We here demonstrate that VALI in patients with normal lungs at the onset of mechanical ventilation is characterized by a procoagulant shift in the bronchoalveolar hemostatic balance. Our results show that it is likely that VALI developed in patients who had had a primary hit. Monitoring coagulation parameters like PAI-1 may be helpful in determining which patients are at risk for VALI.

## Figures and Tables

**Table 1:** Baseline characteristics.

	VALI (*n* = 10)	No VALI (*n* = 10)	*P* value
Age (years)	63 (±13)	55 (±11)	0.13
Gender (male)	7 (70%)	6 (60%)	0.64
Duration of mechanical ventilation before randomization (hours)	21 (±10)	15 (±7)	0.11
APACHE-II score	20 (±8)	22 (±7)	0.43
SOFA	11 (±4)	7 (±4)	0.03
Lung injury score	1.5 (±0.5)	1.2 (±0.8)	0.37
Diagnosis on admittance			
Sepsis	5		
Intracranial hemorrhage		5	
Aortic dissection		1	
Cardiac surgery	1		
Heat stroke		1	
Resuscitation	2	1	
Pneumonia		1	
Trauma	2	1	

APACHE-II: acute physiology and chronic health evaluation-II; SOFA: sepsis organ failure assessment score.
